# Design and Synthesis of Phthalocyanine-Sensitized Titanium Dioxide Photocatalysts: A Dual-Pathway Study

**DOI:** 10.3390/ma18010202

**Published:** 2025-01-05

**Authors:** Qi Shao, Jiaqi Liu, Qiwang Chen, Jing Yu, Zhongbao Luo, Rongqiang Guan, Zichen Lin, Mingxuan Li, Yi Li, Cong Liu, Yan Li

**Affiliations:** 1 School of Electrical and Information, Jilin Engineering Normal University, Changchun 130052, China; 2 School of Metallurgy, Northeastern University, Shenyang 110819, China; 3 School of Materials Science and Engineering, Xiamen University of Technology, Xiamen 361024, China

**Keywords:** photocatalysis, TiO_2_, phthalocyanine, silane coupling agent, direct sensitization

## Abstract

Phthalocyanine-sensitized TiO_2_ significantly enhances photocatalytic performance, but the method of phthalocyanine immobilization also plays a crucial role in its performance. In order to investigate the effect of the binding strategy of phthalocyanine and TiO_2_ on photocatalytic performance, a dual-pathway study has been conducted. On the one hand, zinc-tetra (*N*-carbonylacrylic) aminephthalocyanine (Pc) was directly grafted onto the surface of Fe_3_O_4_@SiO_2_@TiO_2_ (FST). On the other hand, Pc was immobilized on a silane coupling agent ((3-aminopropyl) triethoxysilane) grafted onto the surface of the FST. Through photocatalytic experiments on the two types of composite materials synthesized, the results showed that the photocatalyst obtained by directly sensitizing Pc (FSTP) exhibited better performance on rhodamine B(RhB) removal than did the other photocatalyst using the silane coupling agent (FSTAP). Further mechanistic studies showed that directly sensitized FSTP exhibited more efficient photogenerated electron–hole pair separation, whereas FSTAP linked by a silane coupling agent created an additional transport distance that might greatly affect the photogenerated electron transport. Therefore, the dual-pathway research in this work provides new guidance for efficiently constructing phthalocyanine-sensitized TiO_2_ photocatalysts.

## 1. Introduction

In the face of the increasingly serious water pollution problem [1,2,3,4,5], especially via the organic pollutants that are difficult to decompose, photocatalytic technology provides a green and efficient solution to effectively degrade organic pollutants by converting light energy into chemical energy and generating strong oxidants [6,7,8,9,10]. These strong oxidants can successfully degrade organic pollutants in water, thus providing an effective solution for water pollution treatment.

TiO_2_ is widely used in photocatalysis due to its widely available sources, non-toxicity, and corrosion resistance [11,12,13,14,15,16]. Fe_3_O_4_ exhibits a magnetic response [17], and the magnetic recycling of photocatalysts can be achieved by introducing it into TiO_2_. The introduction of SiO_2_ as an isolating layer can improve the stability and effectively inhibit the recombination of electron–hole pairs when TiO_2_ is combined with Fe_3_O_4_ [18,19]. In addition, TiO_2_ is unable to fully utilize the solar energy due to the low absorption capacity of visible light [20,21,22,23,24,25,26,27]. Phthalocyanine is a hexadecameric ring 18π-electroplanar conjugated molecule consisting of four isoindole substituents linked by nitro atoms [28,29]. Phthalocyanines have attracted much attention in the field of photocatalysis due to their high chemical and thermal stability, visible light absorption, and ease of functional group modification [30,31]. Due to the matching of the band gap structures of TiO_2_ and phthalocyanine, the photocatalytic ability of phthalocyanine-sensitized TiO_2_ can be improved under visible light [32,33,34,35,36].

Zinc-tetra (*N*-carbonylacrylic) aminephthalocyanine (Pc) (Figure S1) is a phthalocyanine derivative prepared from maleic anhydride-modified zinc-tetraamino phthalocyanine, which is soluble in water with the addition of carboxy-functional groups in the side chain of Pc. Pc is able to form a stable structure with the hydroxyl group on the surface of TiO_2_, which enhances the adhesion of phthalocyanine molecules on the surface of TiO_2_. Zeng et al. [34] prepared twin S-scheme photocatalyst of TiO_2_/phthalocyanine nanowire using a self-assembly strategy, which could effectively promote photogenerated electron transfer across multiple heterogeneous interfaces and exhibited remarkable photocatalytic CO_2_ reduction performance, with a CO yield of 237.8 μmol g^−1^h^−1^ under visible light. Bai et al. [36] reported TiO_2_ nanosheets modified with phthalocyanine and Au to obtain wide-spectrum photocatalysts, and ultrasmall Au (3 nm)-mediated photocatalysts showed favorable photoactivity, with a significant increase in the reduction of CO_2_.

However, in our previous work, we found that the photocatalytic cycling stability of phthalocyanine-sensitized TiO_2_ was not adequate, which might be due to the detachment of phthalocyanine molecules [32,35]. In order to overcome this problem, there was an attempt to introduce a silane coupling agent onto the TiO_2_ surface to immobilize the tetracarboxylic phthalocyanine more firmly through chemical bonding. Unexpectedly, this improvement instead led to a further decrease in the photocatalytic performance of the composites. However, instead of using a silane coupling agent as a linker, employing TiO_2_ sensitized directly with phthalocyanine was more effective, which aroused curiosity and necessitated a study of this part of the phenomenon. Therefore, it is necessary to identify the reasons for the performance degradation and explore more effective immobilization methods to maintain and improve the long-term stability and activity of the photocatalysts.

Herein, we report a dual-pathway study to explore the impact of different binding strategies of phthalocyanine-sensitized TiO_2_ on their photocatalytic performance. Firstly, the FSTP photocatalyst was obtained by directly grafting zinc-tetra (N-carbonylacrylic) aminephthalocyanine (Pc) onto the surface of magnetic Fe_3_O_4_@SiO_2_@TiO_2_ (FST). Secondly, Pc was immobilized on (3-aminopropyl) triethoxysilane (APTES) grafted onto the surface of FST to obtain the FSTAP photocatalyst. The results of the photocatalytic experiments showed that the photocatalytic performance of FSTP was better than that of FSTAP in regards to RhB removal. Further mechanistic studies demonstrated that the photogenerated electron–hole pair separation of FSTP was more effective, probably due to the fact that the transport of photogenerated electrons was greatly affected by the transport distance. Therefore, this study provides new guidance for constructing efficient and stable phthalocyanine-sensitized TiO_2_ photocatalysts through optimizing the immobilization methods.

## 2. Materials and Methods

Materials: Fe_3_O_4_ (20 nm) was purchase from Macklin Biochemical Co., Ltd. (Shanghai, China), and the average particle size of Fe_3_O_4_ was 20 nm. Tetraethyl orthosilicate (TEOS), tetrabutyl orthotitanate (TBOT), (3-aminopropyl) triethoxysilane (APTES), *N*-hydroxy succinimide (NHS), sodium sulfide, and 3-ethyl-1(*N,N*-dimethyl)aminopropylcarbodiimide (EDCI) were provided by Beijing Innochem Science & Technology Co., Ltd. (Beijing, China). All other chemicals were used as received, and were not further purified.

Characterization: Fourier transform infrared (FT-IR) spectra were recorded with Bruker OPTICS (Billerica, MA, USA) using an attenuated total reflectance (ATR) accessory with a cumulative total of 32 scans and a nominal resolution of 2 cm^−1^. Thermogravimetric (TG) analysis was recorded using a Netzsch 209F3 thermal gravimetric analyzer (Selb, BY, Germany) at a heating rate of 10 °C/min under N_2_. Scanning electron microscopy (SEM) was performed by a Zeiss EVO 18 device (Oberkochen, BW, Germany) with energy-dispersive spectroscopy (EDS, Oxford X-Maxn). X-ray diffraction (XRD) patterns were recorded on a Rigaku Smartlab X-ray powder diffractometer (Tokyo, Japan) with a wavelength of 0.154 nm. Transmission electron microscopy (TEM) was performed by a JEOL JEM-2100F electron microscope (Tokyo, Japan) with an OXFORD INSTRUMENTS X-Max 80T device. X-ray photoelectron spectra (XPS) were measured with a Kratos AXIS SUPRA + XPS instrument (Tokyo, Japan).

The fabrication of Fe_3_O_4_@SiO_2_ (FS): Pc, FS, and Fe_3_O_4_@SiO_2_@TiO_2_ (FST) was achieved according to the methods detailed in our previous work [35,37]. The synthesis route of Pc was shown in Figure S1. First, zinc tetra-nitrophthalocyanine (TNPc) was synthesized using the solvothermal method at 190 °C for 3 h. Then, TNPc was reduced by sodium sulfide to obtain zinc tetraamino phthalocyanine (TAPc). Finally, the reaction of TAPc with maleic anhydride yielded Pc. First, the Fe_3_O_4_ (0.1 g) was dispersed in a mixed solution containing ethanol (90 mL), deionized water (30 mL), and ammonia (2.5 mL). Then, the solution was sonicated at room temperature for 1 h and under mechanical stirring at 300 r/min. TEOS (0.3 mL) was added drop by drop to the above solution, and stirring was continued at room temperature for 3 h. At the end of the reaction, the products were separated by a magnet and washed several times with deionized water and ethanol. Finally, the samples were dried in a vacuum drying oven at 60 °C for 24 h. 

Fabrication of FST: First, FS (0.05 g) nanoparticles were dispersed into a beaker containing anhydrous ethanol (8 mL) and TBOT (0.04 mL) with sonication for 10 min. Then, the beaker was placed in a 45 mL PTFE liner. A total of 1 mL of deionized water was added to the space between the PTFE liner and the beaker. The reaction was sealed and loaded into a stainless-steel reactor and heated at 150 °C for 12 h. After the reaction was completed and the mixture was cooled to room temperature, the products were separated by a magnet and washed three times repeatedly with distilled water and anhydrous ethanol. Finally, they were dried under vacuum for 24 h at 60 °C.

Fabrication of FSTA: FST nanoparticles (100 mg) were dispersed in methylbenzene (50 mL) for 10 min using ultrasound. Then, APTES (0.04 mL) and triethylamine (0.02 mL) were sequentially added to the above solution and refluxed for 3 h. After the reaction was completed and the mixture was cooled to room temperature, the materials were washed three times with distilled water. Finally, the resulting FSTA was dried at 60 °C under vacuum.

Fabrication of FSTP: FST (50 mg) was dispersed into deionized water (20 mL). Then, Pc (3 mg) was dissolved in dimethyl sulfoxide (1 mL), and the prepared mixture was added dropwise to the FST solution at room temperature with mechanical stirring for 24 h. The obtained FSTP was washed with deionized water and dried at 65 °C for 12 h.

Fabrication of FSTAP: Pc (3 mg) and 3-ethyl-1(*N*,*N*-dimethyl)aminopropylcarbodiimide (EDCI) (0.02 g) were added to deionized water (25 mL). After sonication for 15 min, *N*-hydroxy succinimide (NHS) (0.04 g) was added to the solution, which was stirred for 5 min. Then, 5 mL of aqueous solution containing FSTA (10 mg/mL) was added. After stirring for 24 h at room temperature, the obtained FSTAP was washed with deionized water and dried at 65 °C for 12 h.

Photocatalytic experiments: The rhodamine B(RhB) removal by prepared photocatalysts was carried out using a photocatalytic device (PLS SXE300, Beijing Perfect Light Technology Co., Ltd. (Beijing, China)). The light source was a Xe lamp (300 W) equipped with a visible area bandpass filter (λ > 420 nm). A total of 10 mg of photocatalysts was suspended in the RhB aqueous solution (30 mL, 4.8 mg/L). Before the removal experiments, the suspensions were stirred for 30 min in the dark to reach the equilibrium of adsorption–desorption. The concentration of RhB aqueous solution was analyzed using a UV–Vis 2600 spectrophotometer. The removal of RhB was obtained using the following equation:

(1) $$\;D=\frac{C_{0}-C_{t}}{C_{0}}\times 100\%\;\;\;\;$$

where *C*_0_ and *C_t_* were the initial RhB concentration and the concentration of RhB at *t* hour in the photocatalytic process, respectively.

Photocatalytic cycle test: The photocatalytic cycling experiments for FSTP and FSTAP were analyzed under the optimal experimental parameters. After each degradation experiment, FSTP and FSTAP were separated and recycled with a magnet, washed three times with distilled water to reach a neutral pH, and dried in a vacuum oven at 60 °C for 4 h. Then, FSTP and FSTAP were added to the RhB aqueous solution (30 mL, 4.8 mg/L, pH = 5) to perform the next photocatalytic cycling experiment. The concentration of RhB aqueous solution was analyzed using a UV–Vis 2600 spectrophotometer.

Analysis of active species in photocatalytic processes: The formation of the superoxide radicals (**·**O^2−^) and hydroxyl radicals (**·**OH) generated by FSTP and FSTAP under visible light was analysed using 4-benzoquinone (BQ) and isopropanol (IPA) scavengers. BQ (2 mM) and IPA (10 mM) were employed as scavengers to explore **·**O^2−^ and **·**OH, respectively. IPA and BQ were added to the RhB solution prior to the photocatalytic reaction. The analytical method was the same as that used in the photocatalytic experiment.

## 3. Results and Discussion

**Scheme 1:**
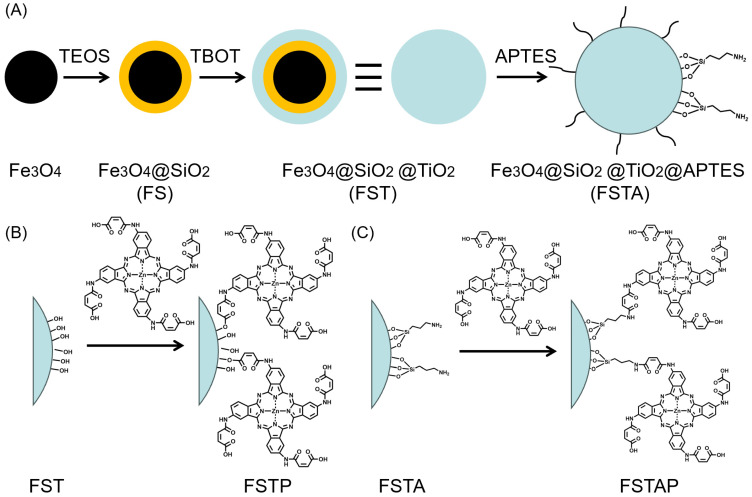
(**A**) Illustration of FST and FSTA synthesis. (**B**) FSTP was prepared by directly grafting Pc onto the surface of FST. (**C**) FSTAP was prepared by Pc immobilized on FSTA with amide linkages.

In this work, two types of photocatalysts for phthalocyanine-sensitized TiO_2_ were prepared, as shown in Scheme 1. As shown in Scheme 1A, firstly, SiO_2_ was coated on Fe_3_O_4_ to obtain Fe_3_O4@SiO_2_ (FS) by the hydrolysis of TEOS; secondly, TiO_2_-coated FS nanoparticles obtained by the hydrolysis of TBOT was used to obtain Fe_3_O_4_@SiO_2_@TiO_2_ (FST); thirdly, FSTA was prepared from (3-aminopropyl)triethoxysilane (APTES)-modified FST. After that, the carboxyl group on Pc was directly combined with the hydroxyl group on magnetic Fe_3_O_4_@SiO_2_@TiO_2_ (FST) to obtain the photocatalyst, namely FSTP (Scheme 1B). In an alternate method, the carboxyl group on Pc was chemically bonded to the amine group on the surface of FSTA to obtain the photocatalyst, namely FSTAP (Scheme 1C). The relationship between the above photocatalysts was explored by evaluating the photocatalytic performance, as well as via structure–photoactivity analysis to further explain the mechanism of phthalocyanine-sensitized TiO_2_.

The structures of the prepared samples were first analyzed using FTIR spectra. Figure S2 showed the FTIR spectra of TNPc, TAPc, and Pc. For TNPc, the characteristic peaks at 1610 cm^−1^ and 1397 cm^−1^ were attributed to the C=N and C–C stretching vibration. The adsorption peaks at 1523 cm^−1^ and 1336 cm^−1^ were attributed to nitro vibration peaks. In the TAPc spectrum, the nitro vibrational peak disappeared, and a new peak at 3329 cm^−1^ was observed, which was attributed to the amine stretching vibration, thus proving that the nitro group in the phthalocyanine was reduced to the amino group. As for Pc, the disappearance of the amine vibrations peak and the appearance of -COOH at 1722 cm^−1^ confirmed the successful preparation of carboxyl-functionalized phthalocyanine. The two peaks at 1075 cm^−1^ and 793 cm^−1^ were assigned to the asymmetric and symmetric vibrations of Si–O–Si in Fe_3_O_4_@SiO_2_ (FS) in Figure S3, respectively. And the peak at 560 cm^−1^ corresponded to the stretching vibration of Fe–O in FS. The peak at 1442 cm^−1^ was attributed to the bending vibration of the C–H bond in FSTA. The peaks at 1652 cm^−1^ and 1360 cm^−1^ were attributed to the vibrations of the -COOTi- group in FSTP. For FSTAP, the peak at 1658 cm^−1^ was attributed to the C=O group of the amide, while the peak at 1572 cm^−1^ corresponded to the N–H bending vibration. Figure S4 shows the XRD patterns of the prepared samples. For FST, the diffraction peaks at 2θ = 29.1°, 34.8°, and 57.8° correspond to (220), (311), and (511), respectively, which were consistent with the PDF# 19-0629 for Fe_3_O_4_. The peaks at 2θ = 25.1°, 48.1°, and 53.8° correspond to (101), (200), and (105), respectively, which belonged to the standard card PDF# 21-1272, suggesting that the material contained TiO_2_, while for FSTP and FSTAP, no significant change was observed.

**Figure 1:**
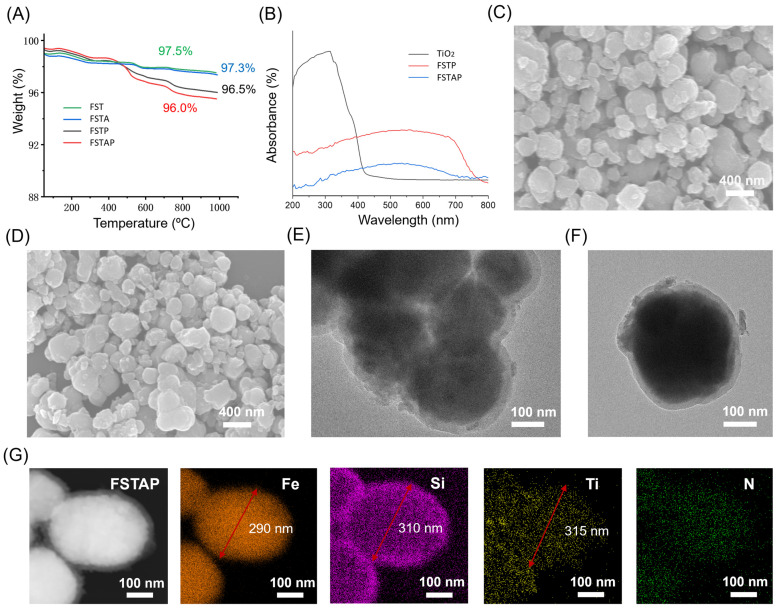
(**A**) TG curves of FST, FSTA, FSTP, and FSTAP. (**B**) UV–Vis spectra of TiO_2_, FSTP, and FSTAP. SEM images of (**C**) FSTP and (**D**) FSTAP. TEM images of (**E**) various and (**F**) single FSTAP microspheres; (**G**) elemental mapping images of FSTAP.

Thermal analysis of the prepared samples was carried out as shown in Figure 1A. From the TG curve, it could be observed that all samples showed a slight weight loss below 100 °C, which was mainly due to the evaporation of adsorbed water molecules on their surfaces. At temperatures between 420 °C and 550 °C, FSTP and FSTAP showed a significant weight loss, which was mainly due to the decomposition of phthalocyanine on the surface of the materials. The weight losses of FST, FSTA, FSTP, and FSTAP were 2.5%, 2.7%, 3.5%, and 4.0% at 1000 °C, respectively. The greater weight loss of FSTAP than FSTP was due to the modification of the FSTAP surface with APTES and the grafting of more Pc. The surface loading of phthalocyanine might be estimated to be about 1% and 1.3% for FSTP and FSTAP. Figure S5 shows the UV–visible absorption spectra of TAPc, TNPc, and Pc, and it was observed that the modified Pc also exhibited absorption in the range of 600–800 nm, similar to that of TAPc and TNPc. Figure 1B further illustrates that TiO_2_ showed no response to visible light and only absorbed UV light, while phthalocyanine-sensitized TiO_2_ enhanced the visible light absorption of the composite. SEM images of Fe_3_O_4_, FS, and FST are shown in Figure S6. Fe_3_O_4_ was uniformly spherical, while FS appeared to aggregate after wrapping on the Fe_3_O_4_ surface. For FST, no significant change was observed, which might be due to low TiO_2_ coating. In Figure 1C,D, the SEM images of FSTP and FSTAP displayed similar round-like structures, with diameters of about 250–350 nm. The elemental mapping images of FSTP and FSTAP, with their EDS patterns, are shown by SEM in Figures S7 and S8. The distribution of the zinc element is observed, which was attributed to the coordination metal in Pc, further indicating that Pc was distributed on the surface of FSTP and FSTAP. TEM images of FSTAP are shown in Figure 1E,F, and the spherical core–shell structure can be clearly observed. The diameters of the distribution range of the Fe, Si, and Ti elements were estimated to be approximately 290 nm, 310 nm, and 315 nm, respectively (Figure 1G). The structures reflected the thicknesses of the SiO_2_ layers and TiO_2_ layers to be roughly 20 nm and 5 nm, respectively.

**Figure 2:**
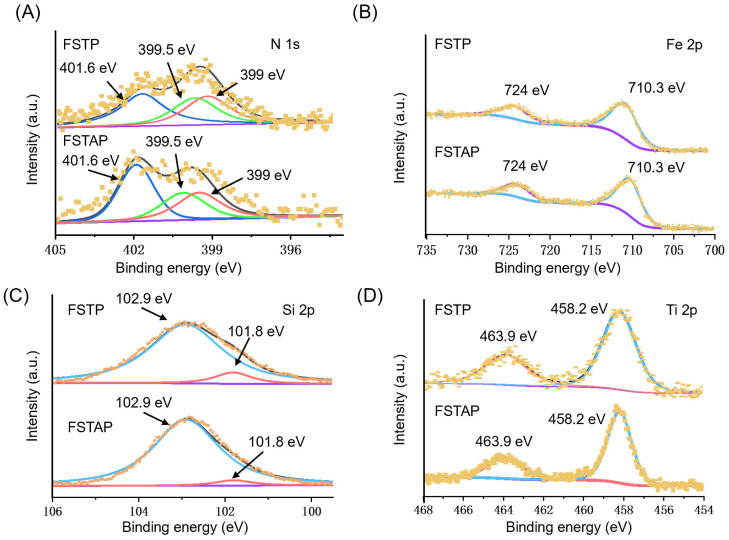
High-resolution XPS spectra of N 1s (**A**), Fe 2p (**B**), Si 2p (**C**), and Ti 2p (**D**) for FSTP and FSTAP.

To analyze the chemical composition and binding environment of the photocatalysts, FSTP and FSTAP were characterized using the X-ray photoelectron spectrum (XPS). The high-resolution XPS spectra of N 1s (A), Fe 2p (B), Si 2p (C), and Ti 2p (D) for FSTP and FSTAP are shown in Figure 2. As for the N 1s fine XPS spectra (Figure 2A), the peaks of FSTP at 399.5 eV and 399 eV were attributed to pyridyl N and pyrrolic N, respectively, which confirmed the Pc on FST. The peak at 401.6 eV could be ascribed to -NH-CO- on Pc [38], while for FSTAP, the peak at 401.6 eV became stronger, which might be caused by the reaction between the carboxyl group of Pc and the amino group on the surface of FSTA to form an amide bond. The two peaks at 724 and 710.3 eV corresponded to the Fe 2p_1/2_ and Fe 2p_3/2_ in Fe 2p (Figure 2B), respectively [39]. This could be ascribed to the detection of Fe elements via the weak points and porous structures in the coatings. The peaks at 102.9 and 101.8 eV were attributed to Si–O–Si and Si(-O)_2_ signals in Si 2p for FSTP and FSTAP (Figure 2C) [40]. The characteristic peaks at 463.9 eV and 458.2 eV in the high-resolution Ti 2p XPS spectra corresponded to Ti 2p_1/2_ and Ti 2p_3/2_ (Figure 2D). The split between Ti 2p_1/2_ and Ti 2p_3/2_ was 5.7 eV, showing that Ti^4+^ existed in FSTP and FSTAP. The element contents of FSTP and FSTAP are shown in Table S1, revealing that the contents of C 1s and N 1s in FSTAP were higher than that of FSTP, which might be caused by the modification of APTES in FSTAP.

**Figure 3:**
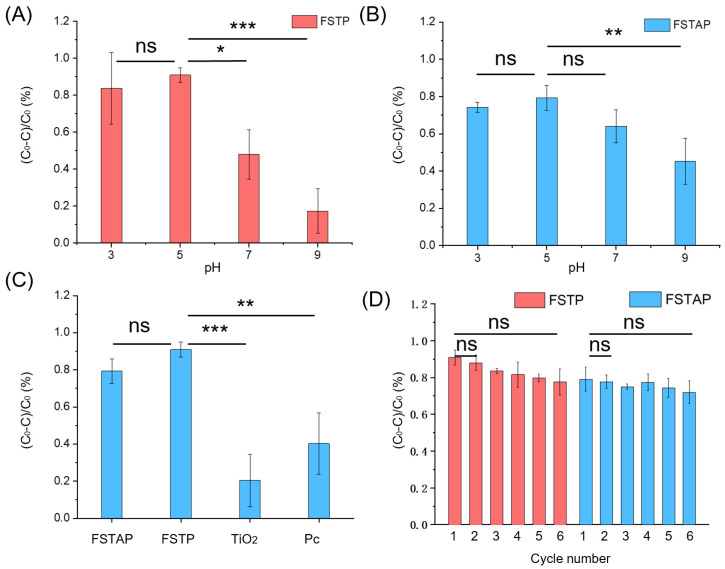
Photocatalytic removal of RhB by (**A**) FSTP and (**B**) FSTAP under visible light at different pH levels in 1 h. (**C**) Photocatalytic removal efficiency of RhB over that of different photocatalysts under visible light at pH = 5 in 1 h. (**D**) Recycling tests of FSTP and FSTAP for RhB removal during six cycles at pH = 5 in 1 h under visible light. Data are presented as mean ± S.D. ns: not significant; * *p* < 0.05; ** *p* < 0.01; *** *p* < 0.001.

The performance of the photocatalysts was evaluated using RhB as the target pollutant. As shown in Figure 3A,B, the photocatalytic removal of RhB by FSTP and FSTAP was investigated under visible light conditions at different pH values within one hour. Sulfuric acid and sodium hydroxide were used to adjust the pH of the solution. FSTP removed more than 80% of RhB in both acidic conditions, while in neutral and alkaline solutions, RhB removal was below 60%. Compared with FSTP, the removal efficiency of RhB was consistently below 80% at different pH levels using FSTAP; the removal efficiency was significantly lower at pH ≥ 7. This might be due to the fact that the acidic conditions helped to enhance the redox reaction activity of the photocatalysts. FSTP and FSTAP achieved 91% and 79% removal rates of RhB at pH = 5, respectively. Data were analyzed through ANOVA test using Graphpad Prism software, with a *p* value of less than 0.05 as the threshold for significance. When FSTP and FSTAP were under acidic conditions, their differences were not significant, but under alkaline conditions, the differences were significant. Figure 3C shows the removal efficiency of RhB with FSTP, FSTAP, TiO_2_, and Pc under visible light at pH = 5 in 1h, corresponding to 91%, 79%, 20%, and 40% removal, respectively, suggesting that the composite photocatalysts with Pc-sensitized TiO_2_ displayed better catalytic properties. It could be observed from the UV–Vis spectra that TiO_2_ displayed no absorption in the visible range. However, the removal of RhB by TiO_2_ reached 20%, which was mainly due to the RhB adsorption on the surface of TiO_2_ with visible light absorption, which in turn sensitized the TiO_2_, making it easier for RhB to self-mineralize [41]. Although the TiO_2_ had the ability to remove RhB, the efficiency was very low, while the phthalocyanine-sensitized TiO_2_ resulted in much improved photocatalytic performance after the addition of FSTP and FSTAP to the solution due to highly efficient photogenerated electron transport. Figure S9 shows the curves of RhB concentration in the solution over time during the photocatalytic process of the prepared samples by UV–Vis spectra. It could be clearly observed that the composites FSTP and FSTAP were effective at removing RhB under acidic conditions. And their photocatalytic performance was superior to that of pure Pc and TiO_2_. Table S2 shows the list of various photocatalysts studied for the removal of RhB, and FSTP displayed an acceptable photocatalytic performance compared to that of other photocatalysts.

In addition, recycling tests of FSTP and FSTAP were conducted for the removal of RhB in 1 h under visible light, and the results are shown in Figure 3D. After six cycles, the photocatalytic performance of FSTP and FSTAP decreased, but the removal efficiency of RhB still reached more than 70%. After six cycles of testing, the removal efficiency of FSTP and FSTAP for RhB decreased by 13% and 7%, respectively. To further illustrate the cycling stability of the materials, Table S3 lists the various photocatalyst cycling tests performed for RhB removal. The prepared photocatalysts demonstrated cycling stability that was comparable to that of the advanced photocatalyst materials recently developed.

**Figure 4:**
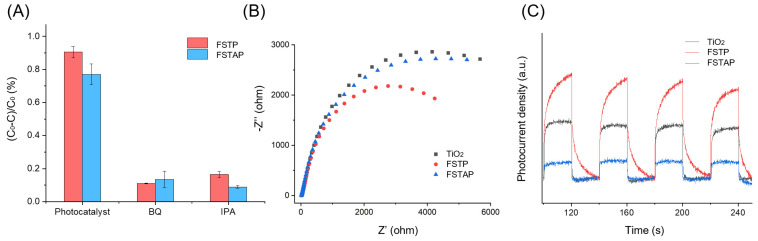
(**A**) Photocatalytic removal efficiency of RhB on FSTP and FSTAP with IPA and BQ, under visible light at 25 °C. (**B**) EIS Nyquist plots and (**C**) transient photocurrents of TiO_2_, FSTP, and FSTAP.

To identify the main active substances for the removal of RhB during the photocatalytic process, Figure 4A shows the addition of BQ and IPA to the photocatalytic experiment to capture **·**O^2−^ and the hydroxyl radicals **·**OH generated during the photocatalytic process, respectively. It shows that the removal rate of RhB was less than 20% in 60 min for both FSTP and FSTAP, indicating that **·**O^2−^ and **·**OH were the active substances in the photocatalytic process. The smaller arc radius of FSTP compared to that of TiO_2_ in Figure 4B indicated the better charge transport performance and the higher separation of the photocatalysts, as well as the transfer efficiency of the photogenerated electrons and holes, which contributed to the efficiency and activity of the photocatalytic reaction. Therefore, FSTP exhibited a lower photogenerated electron transfer resistance. Figure S10 shows the fluorescence intensity in the PL spectra of TiO_2_, FSTP, and FSTAP, and these were also used to explore the recombination ability of the electrons and holes. The emission intensity of FSTP and FSTAP was lower than that of TiO_2_, indicating that FSTP and FSTAP showed superior separation and migration efficiency of the photogenerated electrons to that of TiO_2_. Figure 4C shows the transient photocurrent response measurements (λ > 420 nm) of TiO_2_, FSTP, and FSTAP. Under visible light irradiation, a transient photocurrent for all samples was generated, which immediately increased to a maximum value due to the continuous accumulation of photogenerated electrons on the photocatalyst surface, which rapidly decreased under dark conditions due to the recombination of photogenerated electron carriers. Generally, higher photocurrent intensity meant more efficient separation of the photogenerated charge carriers, which might lead to better FSTP activity than that of FSTAP with silane coupling agents.

The flat band potentials of TiO_2_ and Pc were derived using the Mott–Schottky method as −0.69 V and −1.48 V, respectively (Figures S11 and S12). The conduction band (CB) potentials of TiO_2_ and Pc could be calculated as −0.49 V and −1.28 V by using the equation E_Ag/AgCl_ = E_SHE_ − 0.20. The Pc bandgap value was 2.15 eV, according to our previous work [35]. The band gap value of TiO_2_ was 3.2 eV, which has been reported in previous articles [42,43,44,45,46]. Based on the equation Eg = E_VB_ − E_CB_, the valence band (VB) potentials of TiO_2_ and Pc were 2.71 V and 0.87 V.

**Figure 5:**
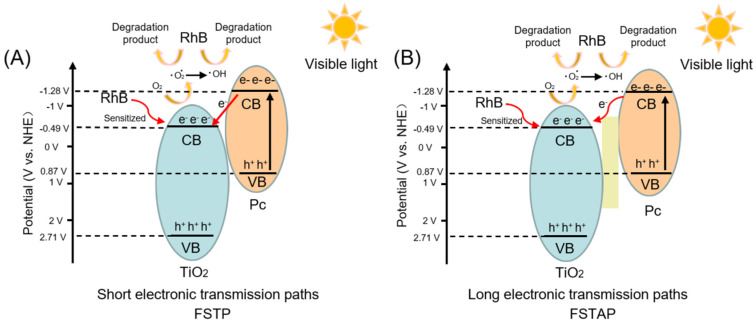
The schematic illustration of the photogenerated electron migration of (**A**) FSTP and (**B**) FSTAP under visible light.

Based on the above experimental analyses, the energy band structure and photogenerated electron transfer of FSTP and FSTAP are shown in Figure 5A,B. Under visible light, Pc generated photogenerated electron–hole pairs, and the photogenerated electrons on the CB of Pc migrated to the CB of TiO_2_, where TiO_2_ was activated, thus effectively prolonging the lifetime of the photogenerated electrons. The photogenerated electrons interacted with dissolved oxygen in water to produce **·**O^2−^. Meanwhile, one electron reduction or disproportionation of **·**O^2−^ resulted in the formation of H_2_O_2_, which could react with the conduction electrons to form the **·**OH [38]. **·**O^2^ and **·**OH were both used for RhB removal, resulting in the effective removal of contaminants from the water. RhB also exhibits the effect of sensitizing TiO_2_ for its own photodegradation. When TiO_2_ was in the RhB solution, RhB absorbed visible light, and photogenerated electron transfer occurred through adsorption on the surface of TiO_2_, leading to the activation of TiO_2_ and the removal of RhB, although the activity of this photocatalyst was low. In the photocatalytic process, the concentration of RhB contained in the solution was the same for FSTP and FSTPA, and it could be assumed that the RhB sensitization effect was comparable. In addition, although the composite of phthalocyanine and TiO_2_ achieved by APTES in FSTAP could increase the loading of Pc, it might also increase the transport distance of the photogenerated electrons generated by Pc to TiO_2_ through APTES, which was likely to directly affect its photocatalytic performance. Therefore, the transmission distance of the photogenerated electrons must be strictly considered when designing composite photocatalysts.

## 4. Conclusions

In summary, phthalocyanine-sensitized TiO_2_ photocatalysts FSTP and FSTAP, with magnetic response, were prepared. FSTP and FSTAP showed a 91% and 79% removal of RhB at pH = 5 under visible light in 1 h, respectively. After six cycles of photocatalytic experiments, the photocatalytic performance of FSTP and FSTAP decreased, but the removal rate of RhB still reached greater than 70%. **·**O^2^ and **·**OH were used for the removal of RhB. Through mechanistic studies, it was also found that the TiO_2_ of FSTP directly sensitized by phthalocyanine contributed more to the enhancement of the photogenerated electron transport than did the silane coupling agent-modified FSTAP. The above findings indicated that the effective contact between the internal components of heterojunction photocatalysts was more favourable for the separation of photogenerated electron–hole pairs, thus improving the photocatalytic performance. This provides valuable references for the design of future photocatalytic systems.

## Data Availability

The original contributions presented in the study are included in the article/Supplementary Material, further inquiries can be directed to the corresponding authors.

## Supplementary Materials

The following supporting information can be downloaded at: https://www.mdpi.com/article/10.3390/ma18010202/s1, Figure S1: Scheme of the Pc synthesis; Figure S2: FTIR spectra of TNPc, TAPc, and Pc; Figure S3: FTIR spectra of FS, FST, FSTA, FSTP, and FSTAP; Figure S4: XRD patterns of FST, FSTP, and FSTAP; Figure S5: UV–Vis spectrum of Pc, TAPc, and TNPc; Figure S6: SEM images of (A) Fe_3_O_4_, (B) FS, and (C) FST; Figure S7: EDS patterns by SEM of FSTP and its elemental mapping images; Figure S8: EDS patterns by SEM of FSTAP and its elemental mapping images; Figure S9: Photocatalytic removal of RhB by FSTP at pH = 3, 5, 7, and 9 under visible light with UV–Vis spectra (A-D); photocatalytic removal of RhB by FSTAP at pH = 3, 5, 7, and 9 under visible light with UV–Vis spectra (E–H); photocatalytic removal of RhB by Pc (I) and TiO_2_ (J) at pH = 5 under visible light with UV–Vis spectra; Figure S10: PL spectra of TiO_2_, FSTP, and FSTAP; Figure S11: Mott–Schottky plots of TiO_2_; Figure S12: Mott–Schottky plots of Pc; Table S1. Atomic content of FSTP and FSTAP by XPS analysis; Table S2: List of various photocatalysts studied for RhB removal; Table S3. List of various photocatalyst cycle tests investigated for RhB removal. References [2,47,48,49,50,51,52,53,54,55] are cited in the supplementary materials

## References

[1] Dai Z., Li D., Chi L., Li Y., Gao B., Qiu N., Duan Q., Li Y. (2019). Preparation of porphyrin sensitized three layers magnetic nanocomposite Fe_3_O_4_@SiO_2_@TiO_2_ as an efficient photocatalyst. Mater. Lett..

[2] Xu T., Wang D., Dong L., Shen H., Lu W., Chen W. (2019). Graphitic carbon nitride comodified by zinc phthalocyanine and graphene quantum dots for the efficient photocatalytic degradation of refractory contaminants. Appl. Catal. B-Environ..

[3] Zhao W., Li J., Dai B., Cheng Z., Xu J., Ma K., Zhang L., Sheng N., Mao G., Wu H. (2019). Simultaneous removal of tetracycline and Cr(VI) by a novel three-dimensional AgI/BiVO4 p-n junction photocatalyst and insight into the photocatalytic mechanism. Chem. Eng. J..

[4] Zhao W., Zhang J., Zhu F., Mu F., Zhang L., Dai B., Xu J., Zhu A., Sun C., Leung D.Y.C. (2019). Study the photocatalytic mechanism of the novel Ag/p-Ag_2_O/nBiVO_4_ plasmonic photocatalyst for the simultaneous removal of BPA and chromium (VI). Chem. Eng. J..

[5] Liang R., Luo S., Jing F., Shen L., Qin N., Wu L. (2015). A simple strategy for fabrication of Pd@MIL-100(Fe) nanocomposite as a visible-light-driven photocatalyst for the treatment of pharmaceuticals and personal care products (PPCPs). Appl. Catal. B-Environ..

[6] Akpan U.G., Hameed B.H. (2009). Parameters affecting the photocatalytic degradation of dyes using TiO_2_-based photocatalysts: A review. J. Hazard. Mater..

[7] Pelaez M., Nolan N.T., Pillai S.C., Seery M.K., Falaras P., Kontos A.G., Dunlop S.M., Hamilton J.W., Byrne J.A., O’Shea K. (2012). A review on the visible light active titanium dioxide photocatalysts for environmental applications. Appl. Catal. B-Environ..

[8] Yang X., Cui H., Li Y., Qin J., Zhang R., Tang H. (2013). Fabrication of Ag_3_PO_4_-Graphene composites with highly efficient and stable visible light photocatalytic performance. ACS Catal..

[9] Yu X., Shavel A., An X., Luo Z., Ibáñez M., Cabot A. (2014). Cu_2_ZnSnS_4_-Pt and Cu_2_ZnSnS_4_-Au heterostructured nanoparticles for photocatalytic water splitting and pollutant degradation. J. Am. Chem. Soc..

[10] Pugazhenthiran N., Murugesan S., Anandan S. (2013). High surface area Ag-TiO_2_ nanotubes for solar/visible-light photocatalytic degradation of ceftiofur sodium. J. Hazard. Mater..

[11] Park S.H., Kim S., Park J.W., Kim S., Cha W., Lee J. (2024). In-situ and wavelength-dependent photocatalytic strain evolution of a single Au nanoparticle on a TiO_2_ film. Nat. Commun..

[12] Zhang W., Deng C., Wang W., Sheng H., Zhao J. (2024). Achieving almost 100% selectivity in photocatalytic CO_2_ reduction to methane via in-situ atmosphere regulation strategy. Adv. Mater..

[13] Liu L., Cui Z., Feng B., Sui M., Huang H., Wu Z. (2024). Synthesis of Fe_2_O_3_/TiO_2_. Photocatalytic composites for methylene blue degradation as a novel strategy for high-value utilisation of iron scales. Materials.

[14] Ghiloufi M., Schnabel T., Mehling S., Kouass S. (2024). Investigation of the effect of oxide additives on the band gap and photocatalytic efficiency of TiO_2_ as a fixed film. Materials.

[15] Ma S., Qin Y., Sun K., Ahmed J., Tian W., Ma Z. (2024). Round-the-clock adsorption-degradation of tetracycline hydrochloride by Ag/Ni-TiO_2_. Materials.

[16] Liao G., Tao X., Fang B. (2022). An innovative synthesis strategy for high-efficiency and defects-switchable-hydrogenated TiO_2_ photocatalysts. Matter.

[17] Yavuz E., Tokalioglu S., Patat S. (2018). Core-shell Fe_3_O_4_ polydopamine nanoparticles as sorbent for magnetic dispersive solid-phase extraction of copper from food samples. Food Chem..

[18] Liu H., Jia Z., Ji S., Zheng Y., Li M., Yang H. (2011). Synthesis of TiO_2_/SiO_2_@Fe_3_O_4_ magnetic microspheres and their properties of photocatalytic degradation dyestuff. Catal. Today.

[19] Gu J., Zhang Q., Li H., Tang Y., Kong J., Dang J. (2007). Study on preparation of SiO_2_/epoxy resin hybrid materials by means of sol-gel. Polym.-Plast. Technol. Eng..

[20] Náfrádi M., Alapi T., Veres B., Farkas L., Bencsik G., Janáky C. (2023). Comparison of TiO_2_ and ZnO for heterogeneous photocatalytic activation of the peroxydisulfate ion in trimethoprim degradation. Materials.

[21] Hua K., Wu Z., Chen W., Xi X., Chen X., Yang S., Gao P., Zheng Y. (2024). Preparation and photocatalytic properties of Al_2_O_3_–SiO_2_–TiO_2_ porous composite semiconductor ceramics. Molecules.

[22] Irodia R., Ungureanu C., Sătulu V., Mîndroiu V.M. (2023). Photocatalyst based on nanostructured TiO_2_ with improved photocatalytic and antibacterial properties. Materials.

[23] Kim H., Jo H., Lee T.S. (2021). Synthesis of chemically bound conjugated polymer on TiO_2_ for a visible-light-driven photocatalyst: Changeable surface wettability. Mater. Deign.

[24] Miyoshi A., Nishioka S., Maeda K. (2018). Water splitting on rutile TiO_2_-based photocatalysts. Chem. Eur. J..

[25] Zhang X., Xiao Y., Cao S., Yin Z., Liu Z. (2022). Ternary TiO_2_@Bi_2_O_3_@TiO_2_ hollow photocatalyst drives robust visible-light photocatalytic performance and excellent recyclability. J. Clean. Prod..

[26] Jiao W., Zhang L., Yang R., Ning J., Xiao L., Liu Y., Ma J., Mahmood N., Jian X. (2022). Synthesis of monolayer carbon-coated TiO_2_ as visible-light-responsive photocatalysts. Appl. Mater. Today.

[27] Chung K.-H., Kim B.-J., Park Y.-K., Kim S.-C., Jung S.-C. (2021). Photocatalytic properties of amorphous N-doped TiO_2_ photocatalyst under visible light irradiation. Catalysts.

[28] Liu D., Ma H., Zhu C., Qiu F., Yu W., Ma L.L., Wei X.W., Han Y.F., Yuan G. (2024). Molecular Co-catalyst confined within a metallacage for enhanced photocatalytic CO_2_ reduction. J. Am. Chem. Soc..

[29] He Z., Liu Y., Li Z., Xu S., Li Z., Bian J., Jing L. (2024). Metal active sites regulation in copper phthalocyanine/bismuth vanadate Z-scheme heterojunction by electron-withdrawing substituent for selective CO_2_ photoreduction in pure water. Appl. Catal. B-Environ..

[30] Miao X., Zhang P., Wang B., Bai X., Liu W. (2024). Bidirectional heterojunctions photocatalyst engineered with separated dual reduction sites for hydrogen evolution. Appl. Catal. B-Environ..

[31] Sun R., Wang Y., Zhang Z., Qu Y., Li Z., Li B., Wu H., Hua X., Zhang S., Zhang F. (2021). Ultrathin phosphate-modulated zinc phthalocyanine/perylenete diimide supermolecule Z-scheme heterojunctions as efficiently wide visible-light photocatalysts for CO_2_ conversion. Chem. Eng. J..

[32] Liu C., Cui X., Li Y., Duan Q. (2020). A hybrid hollow spheres Cu_2_O@TiO_2_-*g*-ZnTAPc with spatially separated structure as an efficient and energy-saving day-night photocatalyst for Cr(VI) reduction and organic pollutants removal. Chem. Eng. J..

[33] Moon H.S., Yong K. (2020). Noble-metal free photocatalytic hydrogen generation of CuPc/TiO_2_ nanoparticles under visible-light irradiation. Appl. Surf. Sci..

[34] Zeng F., Chen Y., Kan L., Liu L., Zhang J., Tian G. (2025). Biphase TiO_2_\Zn-tetracarboxy-phthalocyanine twin S-scheme heterojunction nanowires with efficient charge transfer and CO_2_ photocatalytic conversion. Sep. Purif. Technol..

[35] Liu C., Li Y., Cui X., Liang C., Xing G., Duan Q. (2021). Construction of a recyclable dual-responsive TiO_2_-based photocatalyst modified with ZnIn_2_S_4_ nanosheets and zinc phthalocyanine for Cr(VI) reduction under visible light. Chem. Eng. J..

[36] Bai L., Li W., Chu X., Yin H., Qu Y., Kozlova E., Yang Z., Jing L. (2025). Effects of nanosized Au on the interface of zinc phthalocyanine/TiO_2_ for CO_2_ photoreduction. Chin. Chem. Lett..

[37] Liu C., Li Y., Duan Q. (2020). Preparation of magnetic and thermal dual-responsive zinc-tetracarboxyl-phthalocyanine-*g*-Fe_3_O_4_@SiO_2_@TiO_2_-*g*-poly(*N*-isopropyl acrylamide) core-shell green photocatalyst. Appl. Surf. Sci..

[38] Lu W., Xu T., Wang Y., Hu H., Li N., Jiang X., Chen W. (2016). Synergistic photocatalytic properties and mechanism of *g*-C_3_N_4_ coupled with zinc phthalocyanine catalyst under visible light irradiation. Appl. Catal. B-Environ..

[39] Xing Y., Ma F., Peng M., Ma X., Zhang Y., Cui Y. (2019). Bifunctional sodium tartrate as stabilizer and reductant for the facile synthesis of Fe_3_O_4_/Ag nanocomposites with catalytic activity. J. Magn. Magn. Mater..

[40] Wei Z., Ma X., Zhang Y., Guo Y., Wang W., Jiang Z. (2022). High-efficiency adsorption of phenanthrene by Fe_3_O_4_-SiO_2_-dimethoxydiphenylsilane nanocomposite: Experimental and theoretical study. J. Hazard. Mater..

[41] Amalia F., Takashima M., Ohtani B. (2022). Are you still using organic dyes? Colorimetric formaldehyde analysis for true photocatalytic-activity evaluation. Chem. Commun..

[42] Khan H., Shah M.U.H. (2023). Modification strategies of TiO_2_ based photocatalysts for enhanced visible light activity and energy storage ability: A review. J. Environ. Chem. Eng..

[43] Cho S., Ahn C., Park J., Jeon S. (2018). 3D nanostructured N-doped TiO_2_ photocatalysts with enhanced visible absorption. Nanoscale.

[44] Zhao Z., Long Y., Luo S., Wu W., Ma J. (2019). Preparation of a magnetic mesoporous Fe_3_O_4_–Pd@TiO_2_ photocatalyst for the efficient selective reduction of aromatic cyanides. New J. Chem..

[45] Wu F., Chen J., Yang Z. (2022). Preparation of C/Ho co-doped TiO_2_ for enhancing the photocatalytic degradation efficiency of tetracycline hydrochloride. New J. Chem..

[46] Makuła P., Pacia M., Macyk W. (2018). How to correctly determine the band gap energy of modified semiconductor photocatalysts based on UV–Vis spectra. J. Phys. Chem. Lett..

[47] Wu Y., Wang H., Tu W., Wu S., Liu Y., Tan Y.Z., Luo H., Yuan X., Chew J.W. (2018). Petal-like CdS nanostructures coated with exfoliated sulfur-doped carbon nitride via chemically activated chain termination for enhanced visible-light–driven photocatalytic water purification and H_2_ generation. Appl. Catal. B-Environ..

[48] Cai Y., Song J., Liu X., Yin X., Li X., Yu J., Ding B. (2018). Soft BiOBr@TiO_2_ nanofibrous membranes with hierarchical heterostructures as efficient and recyclable visible-light photocatalysts. Environ. Sci.-Nano.

[49] Feng Q., Tang D., Lv H., Zhang W., Li W. (2017). Surface-initiated ATRP to modify ZnO nanoparticles with poly(*N*-isopropylacrylamide): Temperature-controlled switching of photocatalysis. J. Alloys Compd..

[50] Cai L., Li Y., Li Y., Wang H., Yu Y., Liu Y., Duan Q. (2018). Synthesis of zincphthalocyanine-based conjugated microporous polymers with rigid-linker as novel and green heterogeneous photocatalysts. J. Hazard. Mater..

[51] Luo J., Liu X., Gu J., Zhao W., Gu M., Xie Y. (2024). Construction of novel g-C_3_N_4_/β-FeOOH Z-Scheme heterostructure photocatalyst modified with carbon quantum dots for efficient degradation of RhB. J. Mater. Sci. Technol..

[52] Sang Y., Cao X., Dai G., Wang L., Peng Y., Geng B. (2020). Facile one-pot synthesis of novel hierarchical Bi_2_O_3_/Bi_2_S_3_ nanoflower photocatalyst with intrinsic p-n junction for efficient photocatalytic removals of RhB and Cr(VI). J. Hazard. Mater..

[53] Ma H., Wang Y., Zhang Z., Liu J., Yu Y., Zuo S., Li B. (2023). A superior ternary Z-scheme photocatalyst of Bi/Black Phosphorus nanosheets/P-doped BiOCl containing interfacial P–P bond and metallic mediator for H_2_O_2_ production and RhB degradation. Chemosphere.

[54] Zhao J., Guo T., Wang H., Yan M., Qi Y. (2023). BiOBr nanoflakes with engineered thickness for boosted photodegradation of RhB under visible light irradiation. J. Alloys Compd..

[55] Li Y., Zheng X., Yang J., Zhao Z., Cui S. (2021). Enhanced photocatalytic degradation of 2,4,6-trichlorophenol and RhB with RhB-sensitized BiOClBr catalyst based on response surface methodology. J. Taiwan Inst. Chem. Eng..

