# Supplementary material for: Design and Synthesis of Phthalocyanine-Sensitized Titanium Dioxide Photocatalysts: A Dual-Pathway Study
Source: Materials (Basel). 2025 Jan 5;18(1):202. doi: 10.3390/ma18010202 (PMC11722483; doi:10.3390/ma18010202)
Supplement: Supplementary file 1 [file materials-18-00202-s001.zip › materials-3400313-supplementary.pdf]

materials

Supporting Information

Design and Synthesis of Phthalocyanine-Sensitized Titanium Dioxide Photocatalysts: A Dual-Pathway Study

Qi Shao [1], Jiaqi Liu [2], Qiwang Chen [1], Jing Yu [1], Zhongbao Luo [1], Rongqiang Guan [1], Zichen Lin [3], Mingxuan Li [3], Yi Li [1,*], Cong Liu [3,*] and Yan Li [1]

[1] School of Electrical and Information, Jilin Engineering Normal University, Changchun 130052, China
[2] School of Metallurgy, Northeastern University, Shenyang 110819, China
[3] School of Materials Science and Engineering, Xiamen University of Technology, Xiamen 361024, China
* Correspondence: liyi6929@163.com (Y.L.); cuigu808@126.com (C.L.)

Supplemental Figures

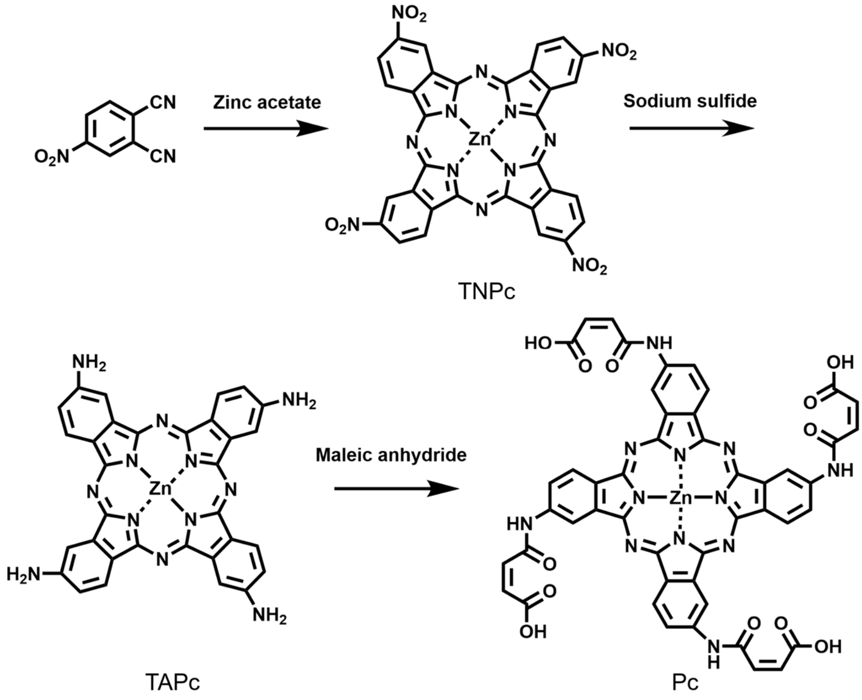

Figure S1. Scheme of the Pc synthesis.

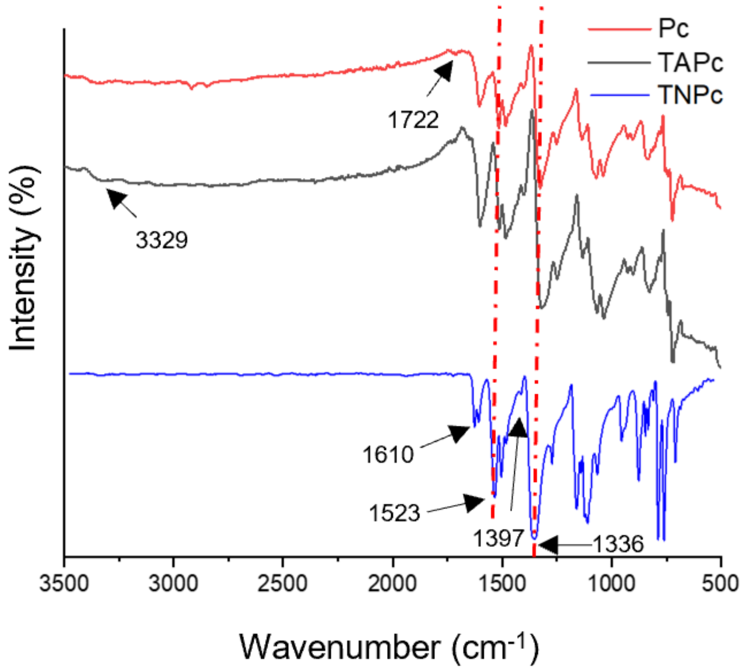

Figure S2. FT-IR spectra of TNPc, TAPc and Pc.

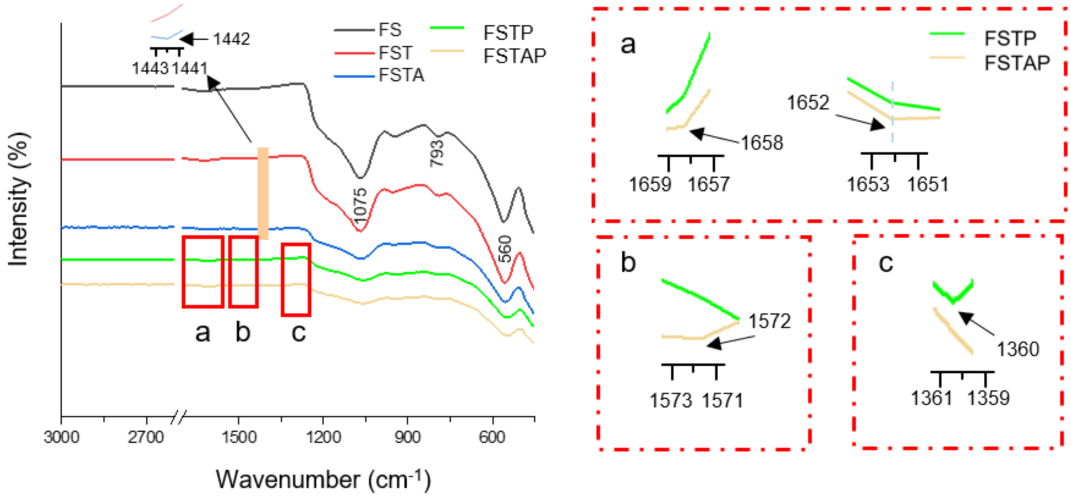

Figure S3. FT-IR spectra of FS, FST, FSTA, FSTP and FSTAP.

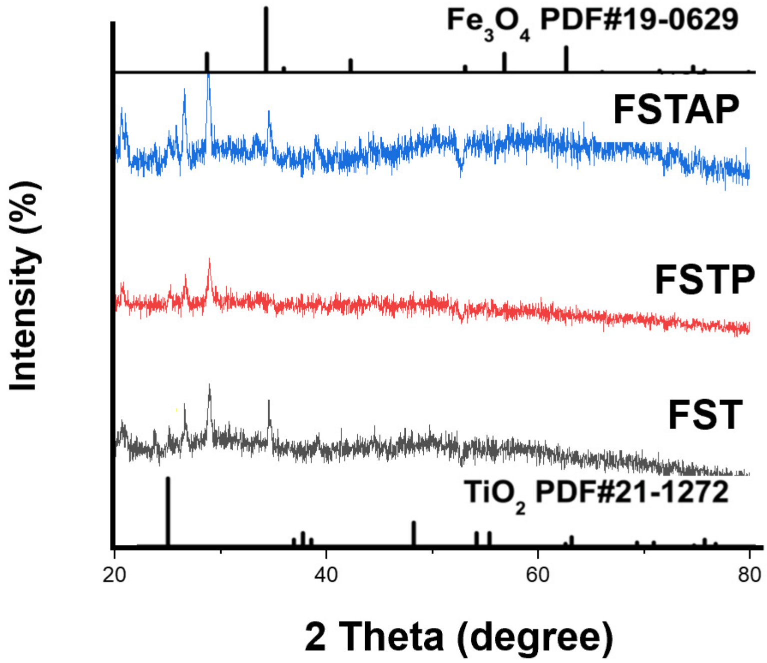

Figure S4. XRD patterns of FST, FSTP and FSTAP.

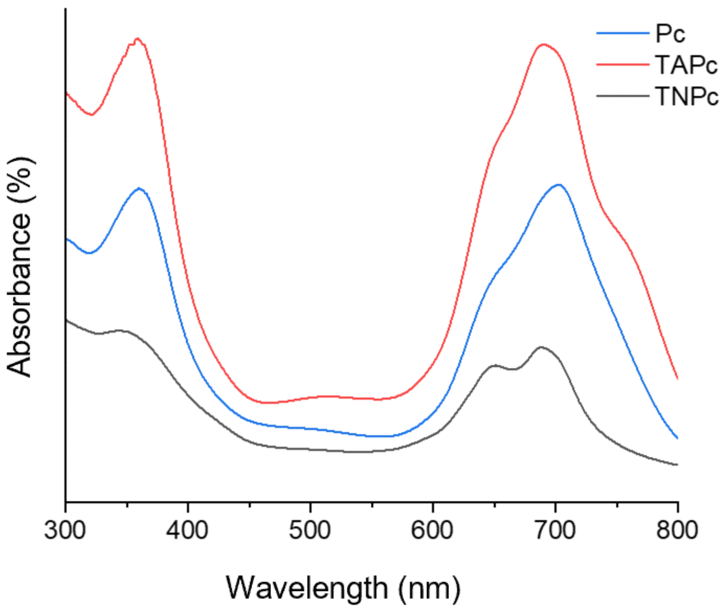

Figure S5. UV-Vis spectrum of Pc, TAPc and TNPc.

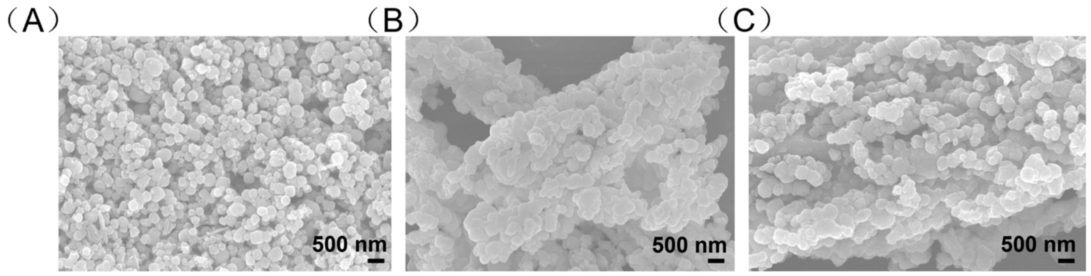

Figure S6. SEM images of (A) Fe_3O_4, (B) FS and (C) FST.

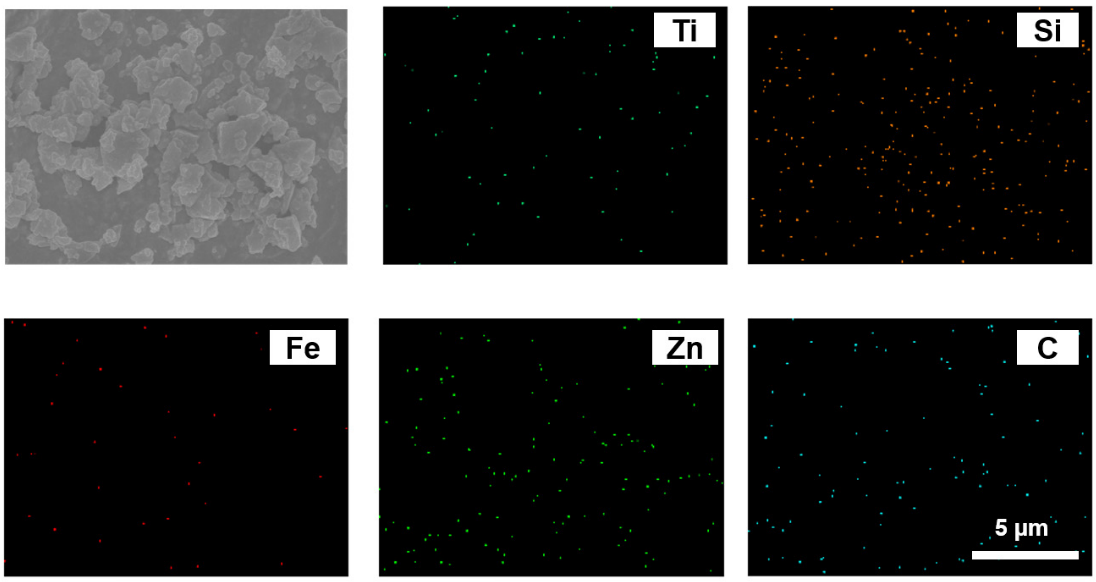

Figure S7. EDS patterns by SEM of FSTP and its elemental mapping images.

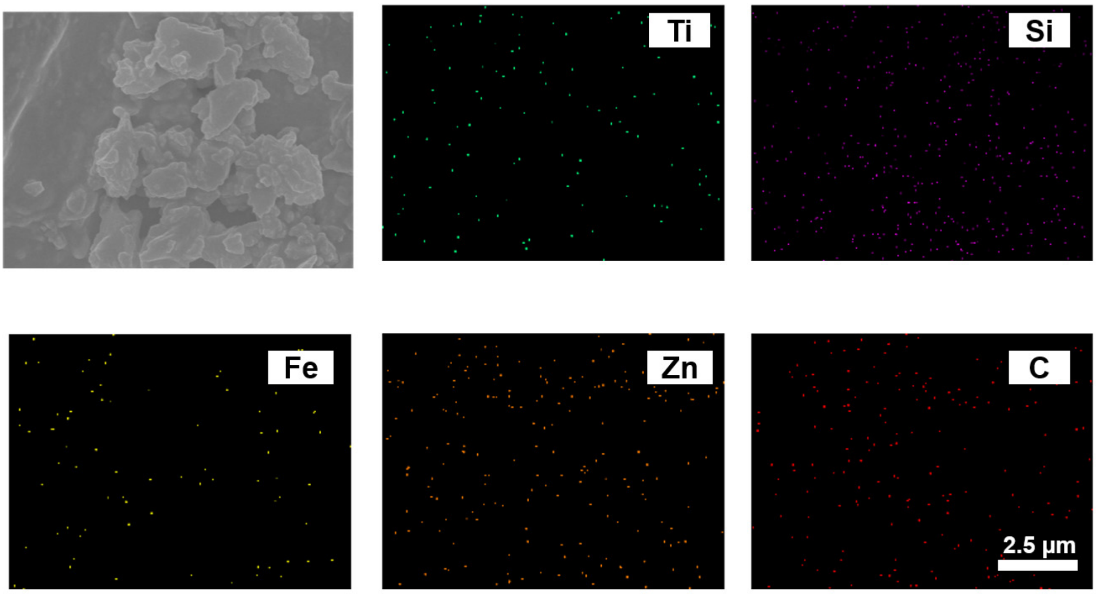

Figure S8. EDS patterns by SEM of FSTAP and its elemental mapping images.

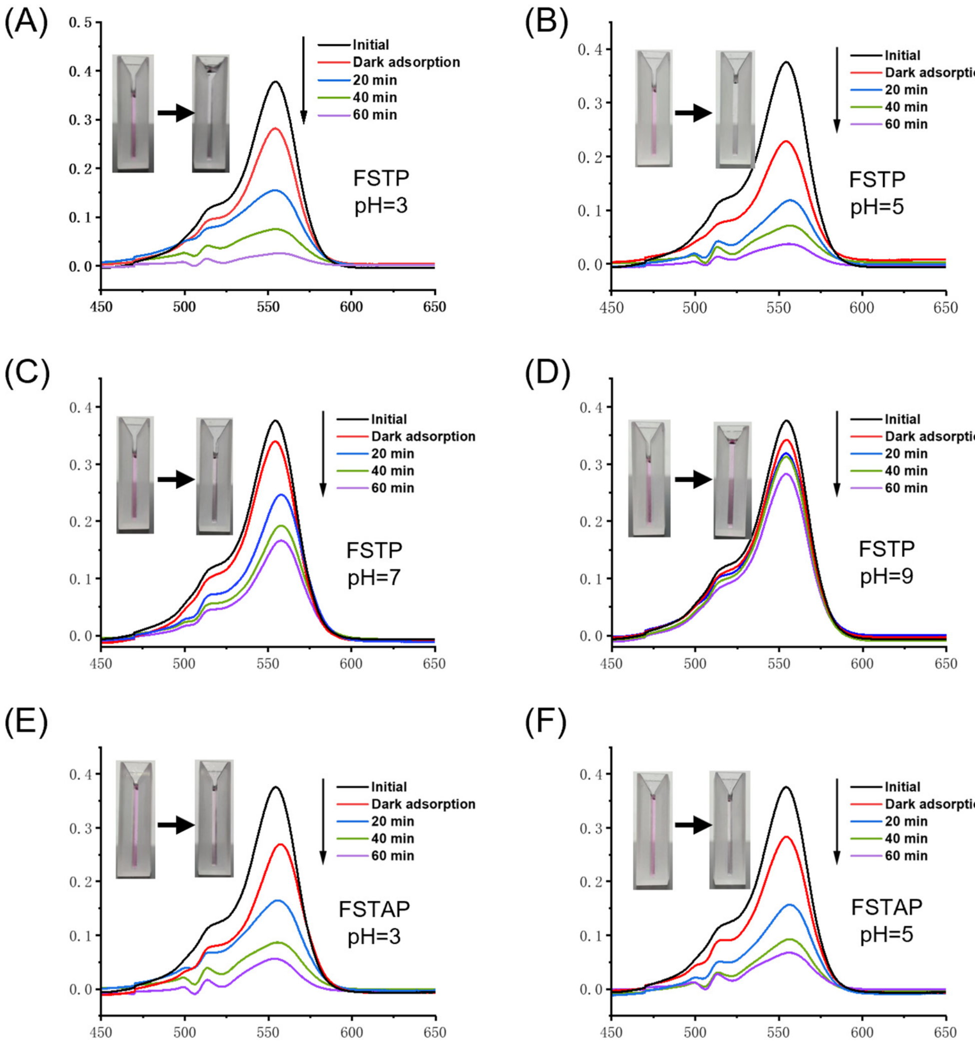

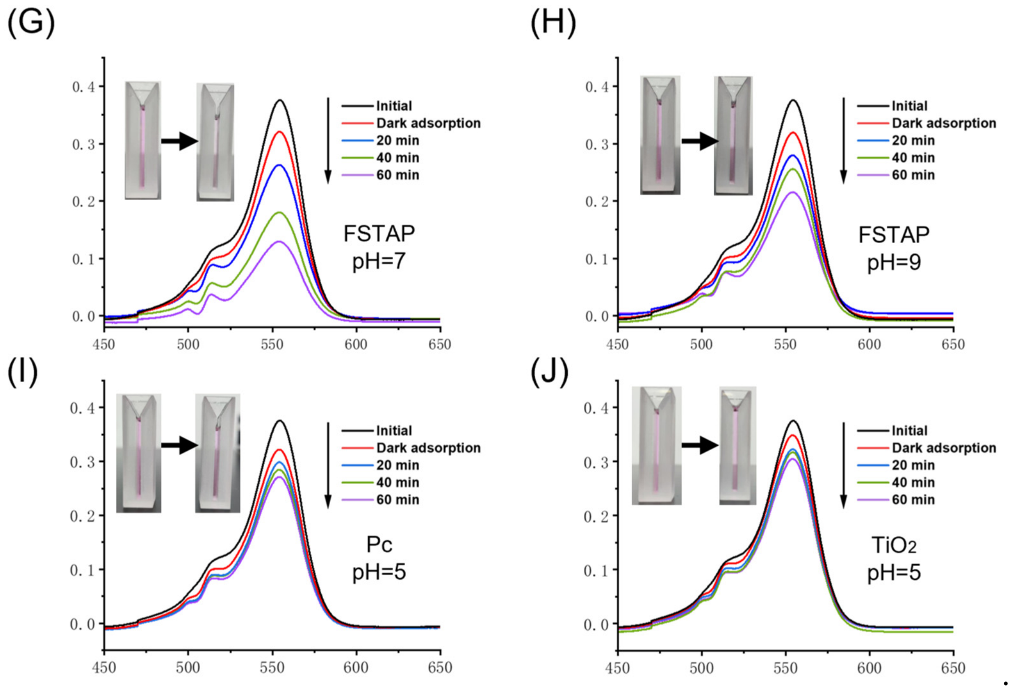

Figure S9. Photocatalytic removal of RhB by FSTP at pH=3, 5, 7, 9 under visible light with UV-Vis spectra (A-D); photocatalytic removal of RhB by FSTAP at pH=3, 5, 7, 9 under visible light with UV-Vis spectra (E-H); photocatalytic removal of RhB by Pc (I) and TiO_2 (J) at pH= 5 under visible light with UV-Vis spectra.

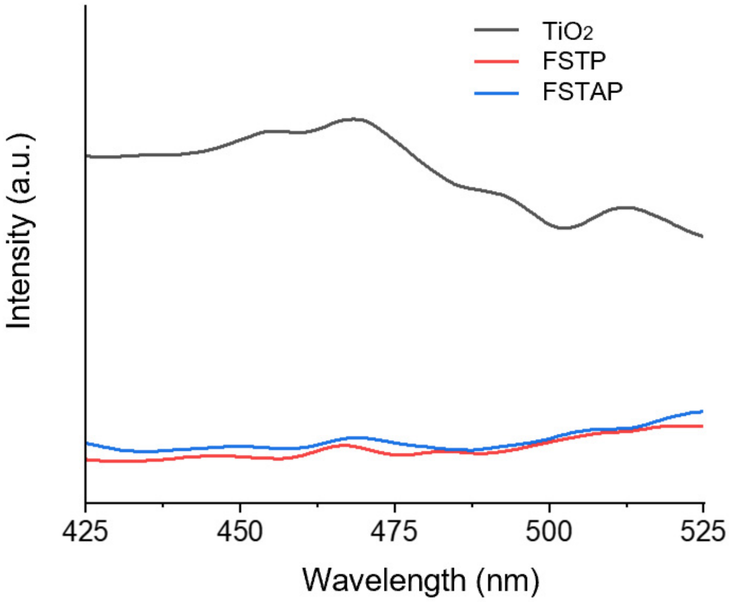

Figure S10. PL spectra of TiO_2, FSTP and FSTAP (excitation: 315 nm).

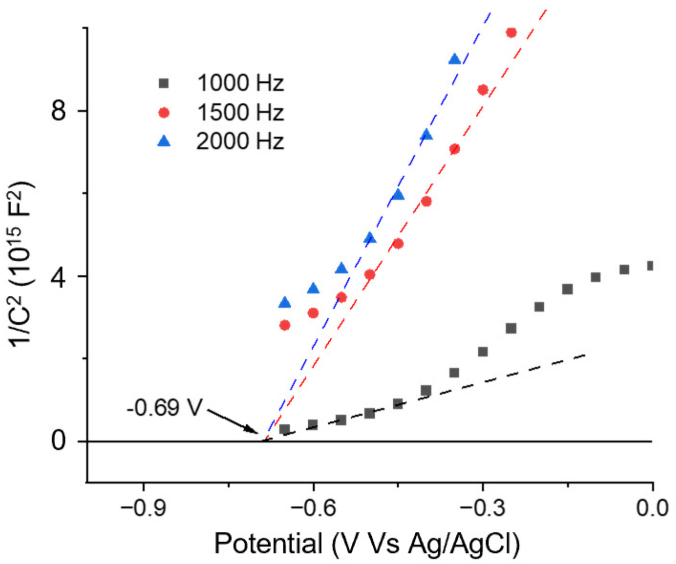

Figure S11. Mott-Schottky plots of TiO_2.

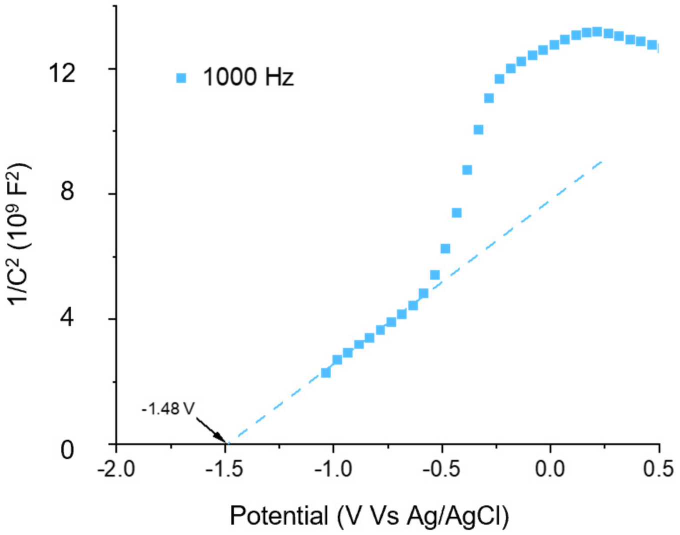

Figure S12. Mott-Schottky plots of Pc.

Table S1. Atomic content of FSTP and FSTAP by XPS analysis.

Compound	Atomic conc. [%] FSTP	Atomic conc. [%] FSTAP
O 1s	52.4	51.62
C 1s	22.9	23.61
Si 2p	19.08	18.84
Fe 2p	3.34	3.34
N 1s	1.47	1.7
Ti 2p	0.81	0.89

Table S2. List of various photocatalysts studied for RhB removal.

Sample	Catalyst mass (mg)	Efficiency of RhB removal	Time	Ref
FSTP	10	90%	60 min	Our work
g-C_3N_4/ZnTcPc/GQDs	2	73%	60 min	[1]
CdS/SCN	50	about 100%	90 min	[2]
BiOBr@TiO_2	2	95.5%	120 min	[3]
ZnO-PNIPAM	20	10.05 %	60 min	[4]
ZnPc-CMP	10	98 %	180min	[5]

Table S3. List of various photocatalyst cycle tests investigated for RhB removal.

Sample	Cycle number	Decrease in RhB degradation	Ref
FSTP	6	13%	Our work
FSTAP	6	7%	Our work
g-C_3N_4/β-FeOOH	5	about 20%	[6]
Bi_2O_3/Bi_2S_3	5	14%	[7]
Bi/BPNs/P–BiOCl-20	6	about 0%	[8]
BiOBr nanoflakes	5	12%	[9]
BiOClBr	5	about 15 %	[10]

References

1. Xu, T.; Wang, D.; Dong, L.; Shen, H.; Lu, W.; Chen, W. Graphitic carbon nitride co-modified by zinc phthalocyanine and graphene quantum dots for the efficient photocatalytic degradation of refractory contaminants. *Appl. Catal. B-Environ.* **2019**, *244*, 96–106. https://doi.org/10.1016/j.apcatb.2018.11.049.
2. Wu, Y.; Wang, H.; Tu, W.; Wu, S.; Liu, Y.; Tan, Y.Z.; Luo, H.; Yuan, X.; Chew, J.W. Petal-like CdS nanostructures coated with exfoliated sulfur-doped carbon nitride via chemically activated chain termination for enhanced visible-light–driven photocatalytic water purification and H_2 generation. *Appl. Catal. B-Environ.* **2018**, *229*, 181–191. https://doi.org/10.1016/j.apcatb.2018.02.029.
3. Cai, Y.; Song, J.; Liu, X.; Yin, X.; Li, X.; Yu, J.; Ding, B. Soft BiOBr@TiO_2 nanofibrous membranes with hierarchical heterostructures as efficient and recyclable visible-light photocatalysts. *Environ. Sci.-Nano* **2018**, *5*, 2631–2640. https://doi.org/10.1039/C8EN00866C.
4. Feng, Q.; Tang, D.; Lv, H.; Zhang, W.; Li, W. Surface-initiated ATRP to modify ZnO nanoparticles with poly(*N*-isopropylacrylamide): Temperature-controlled switching of photocatalysis. *J. Alloys Compd.* **2017**, *691*, 185–194. https://doi.org/10.1016/j.jallcom.2016.08.226.
5. Cai, L.; Li, Y.; Li, Y.; Wang, H.; Yu, Y.; Liu, Y.; Duan, Q. Synthesis of zincphthalocyanine-based conjugated microporous polymers with rigid-linker as novel and green heterogeneous photocatalysts. *J. Hazard. Mater.* **2018**, *345*, 47–55. https://doi.org/10.1016/j.jhazmat.2018.01.027.
6. Luo, J.; Liu, X.; Gu, J.; Zhao, W.; Gu, M.; Xie, Y. Construction of novel g-C_3N_4/β-FeOOH Z-Scheme heterostructure photocatalyst modified with carbon quantum dots for efficient degradation of RhB. *J. Mater. Sci. Technol.* **2024**, *181*, 11–19. https://doi.org/10.1016/j.jmst.2023.10.006.
7. Sang, Y.; Cao, X.; Dai, G.; Wang, L.; Peng, Y.; Geng, B. Facile one-pot synthesis of novel hierarchical Bi_2O_3/Bi_2S_3 nanoflower photocatalyst with intrinsic p-n junction for efficient photocatalytic removals of RhB and Cr(VI). *J. Hazard. Mater.* **2020**, *381*, 120942. https://doi.org/10.1016/j.jhazmat.2019.120942.
8. Ma, H.; Wang, Y.; Zhang, Z.; Liu, J.; Yu, Y.; Zuo, S.; Li, B. A superior ternary Z-scheme photocatalyst of Bi/Black Phosphorus nanosheets/P-doped BiOCl containing interfacial P–P bond and metallic mediator for H_2O_2 production and RhB degradation. *Chemosphere* **2023**, *330*, 138717. https://doi.org/10.1016/j.chemosphere.2023.138717.
9. Zhao, J.; Guo, T.; Wang, H.; Yan, M.; Qi, Y. BiOBr nanoflakes with engineered thickness for boosted photodegradation of RhB under visible light irradiation. *J. Alloys Compd.* **2023**, *947*, 169613. https://doi.org/10.1016/j.jallcom.2023.169613.

10. Li, Y.; Zheng, X.; Yang, J.; Zhao, Z.; Cui, S. Enhanced photocatalytic degradation of 2,4,6-trichlorophenol and RhB with RhB-sensitized BiOClBr catalyst based on response surface methodology. *J. Taiwan Inst. Chem. Eng.* **2021**, *119*, 213–223. https://doi.org/10.1016/j.jtice.2021.02.014.
